# The Burden of Typhoid and Paratyphoid in India: Systematic Review and Meta-analysis

**DOI:** 10.1371/journal.pntd.0004616

**Published:** 2016-04-15

**Authors:** Jacob John, Carola J. C. Van Aart, Nicholas C. Grassly

**Affiliations:** 1 Department of Community Health, Christian Medical College, Vellore, India; 2 Department of Infectious Disease Epidemiology, Imperial College London, London, United Kingdom; Oxford University Clinical Research Unit, VIETNAM

## Abstract

**Background:**

Typhoid is an important public health challenge for India, especially with the spread of antimicrobial resistance. The decision about whether to introduce a public vaccination programme needs to be based on an understanding of disease burden and the age-groups and geographic areas at risk.

**Methods:**

We searched Medline and Web of Science databases for studies reporting the incidence or prevalence of typhoid and paratyphoid fever confirmed by culture and/or serology, conducted in India and published between 1950 and 2015. We used binomial and Poisson mixed-effects meta-regression models to estimate prevalence and incidence from hospital and community studies, and to identify risk-factors.

**Results:**

We identified 791 titles and abstracts, and included 37 studies of typhoid and 18 studies of paratyphoid in the systematic review and meta-analysis. The estimated prevalence of laboratory-confirmed typhoid and paratyphoid among individuals with fever across all hospital studies was 9.7% (95% CI: 5.7–16.0%) and 0.9% (0.5–1.7%) respectively. There was significant heterogeneity among studies (p-values<0.001). Typhoid was more likely to be detected among clinically suspected cases or during outbreaks and showed a significant decline in prevalence over time (odds ratio for each yearly increase in study date was 0.96 (0.92–0.99) in the multivariate meta-regression model). Paratyphoid did not show any trend over time and there was no clear association with risk-factors. Incidence of typhoid and paratyphoid was reported in 3 and 2 community cohort studies respectively (in Kolkata and Delhi, or Kolkata alone). Pooled estimates of incidence were 377 (178–801) and 105 (74–148) per 100,000 person years respectively, with significant heterogeneity between locations for typhoid (p<0.001). Children 2–4 years old had the highest incidence.

**Conclusions:**

Typhoid remains a significant burden in India, particularly among young children, despite apparent declines in prevalence. Infant immunisation with newly-licensed conjugate vaccines could address this challenge.

## Author Summary

Typhoid fever is an important cause of avoidable mortality in regions without adequate access to safe water and sanitation. Highly immunogenic typhoid conjugate vaccines are now licensed and under consideration as a public health intervention in India. The decision about whether and how to introduce a public vaccination programme needs to be based on an understanding of disease burden, and the age-groups and geographic areas at risk. We performed a systematic review and meta-analysis of published studies reporting typhoid and paratyphoid incidence and prevalence in India between 1950 and 2015. The estimated prevalence of laboratory-confirmed typhoid and paratyphoid among individuals with fever across all hospital studies was 9.7% (95% CI: 5.7–16.0%) and 0.9% (0.5–1.7%) respectively, with a significant decline in prevalence of the former over time. We found only three population-based studies that reported incidence. Pooled estimates were 377 (178–801) and 105 (74–148) per 100,000 person years for typhoid and paratyphoid respectively, with incidence being highest in in children between 2 and 4 years. Despite an apparent decline in prevalence, typhoid remains a significant burden in India, particularly among young children. Studies are required to evaluate the effectiveness of infant immunisation with conjugate typhoid vaccines.

## Introduction

Typhoid (enteric) fever caused by *Salmonella enterica* serovar Typhi (*S*. Typhi) is an important cause of morbidity and mortality. The global annual burden was estimated at approximately 12 million cases for 2010 [1,2]. Most of these were effectively treated with antibiotics, although the case fatality rate remains at about 1% such that about 130,000 typhoid deaths occur annually. Antibiotic resistance is a challenge for effective treatment of typhoid and is likely to become increasingly problematic with the spread of multi-drug resistant strains [3]. The situation is further complicated by increased incidence in some countries of *S*. Paratyphi A as a cause of enteric fever [4]. This serovar is not prevented by currently available typhoid vaccines and represents an increasing threat to human health.

The incidence of typhoid and paratyphoid varies geographically, with south-central and south-east Asia having the highest incidence—typically exceeding 100 cases per 100,000 person-years for typhoid and with lower, variable rates for paratyphoid. In one multicenter study, the annual incidence of typhoid per 100,000 children aged 5–15 years was 180 in North Jakarta, Indonesia, 413 in Karachi, Pakistan and 494 in Kolkata, India [5]. In the same settings, the annual incidence of paratyphoid was considerably lower, with the highest annual incidence reported from Pakistan of 72 per 100,000 children aged 2–16 years [6].

The burden of typhoid fever shows substantial variation within as well as between countries. Commonly identified risk-factors include a lack of clean drinking water, poor sanitation, inadequate hygiene practices and low socio-economic status [2,7]. Outbreaks may occur following a defined event of food or water contamination with the bacterium, in which case locally-specific risk factors or exposures may be identified e.g. eating milk products from a sweet shop, [8]. In some instances the originating infection may be a chronic carrier who persistently sheds the bacterium as a result of infection of the gall bladder. Chronic carriage occurs following primary infection in approximately 2–5% of cases in the absence of antibiotic treatment and is strongly dependent on age and sex [9]. However, the contribution of chronic carriers to typhoid transmission in endemic regions is unknown [10].

Several safe and effective vaccines that could help reduce disease burden are licensed and available in India. Three or four doses of orally-administered, live-attenuated Ty21a provide about 50–70% protection for at least 7 years and are licensed in capsule form from 5 years of age or as a liquid formulation from 2 years of age [11,12,13]. The single-dose injectable Vi polysaccharide vaccine provides similar levels of protection for at least 3 years and is licensed from 2 years of age [11,14,15]. A Vi polysaccharide conjugated to P*seudomonas aeruginosa* exotoxin A (rEPA) as a carrier protein and administered to 2–5 year old children gave approximately 90% protective efficacy against typhoid over 4 years [16,17]. More recently, two Vi polysaccharide vaccines conjugated to tetanus toxoid have been licensed in India from 3–6 months of age based on their encouraging immunogenicity [18]. The immunogenicity of conjugate typhoid vaccines in children under 2 years of age (cf. Vi polysaccharide vaccines) is an important advance, given the significant burden of disease in young children and infants [19].

The WHO recommends the programmatic use of typhoid vaccines for controlling endemic disease, although in most countries vaccinating only high risk populations is recommended [20]. In India, routine typhoid vaccination is not implemented and decision-making has been hampered by the lack of reliable disease burden data with very few prospective surveillance studies in the past two decades. The one exception we are aware of is in Delhi where each year approximately 300,000 children aged 2–5 years are vaccinated with Vi polysaccharide vaccine. With the recent development of conjugate vaccines that can be administered to children under 2 years old, the case for more widespread immunization is stronger and in 2014 the Indian Academy of Pediatrics (IAP) Advisory Committee on Vaccines and Immunization Practices (ACVIP) strongly urged the Government of India (GoI) “to include universal typhoid vaccination in its UIP [Universal Immunisation Programme] all over the country.” [21]

The GoI decisions about whether to introduce a public typhoid vaccination program, its extent and the immunization schedule, need to be based on a firm understanding of the disease burden and the age-groups and geographic areas at risk. We therefore carried out a systematic review to estimate the burden of typhoid and paratyphoid in India and to identify knowledge gaps that need further evaluation. We searched for hospital and community-based studies that reported the incidence or prevalence of typhoid and paratyphoid fever and used meta-analysis and meta-regression models to summarize our findings and identify risk factors for disease.

## Methods

### Systematic review

We searched Medline and the Web of Science literature databases for articles published between 1950 and May 2015 for studies on the burden of typhoid or paratyphoid fever in India, with no language restriction. The search consisted of terms related to typhoid or paratyphoid fever (typhoid OR Salmonella Typhi OR enteric fever OR Salmonella enterica OR paratyphoid OR Paratyphi), combined with terms for Indian geography (including a list of state names) and terms for measures of incidence and prevalence (burden OR incidence OR prevalence OR mortality, etc.). The complete search term is given in the S1 Appendix.

Titles and abstracts of articles were read to identify potentially relevant articles. Studies were considered eligible for further examination in full text if they reported incidence, prevalence, number of reported cases, mortality or the burden of typhoid or paratyphoid in India. Studies were also examined in full text if only a title was returned by the initial search. Full text articles were obtained through online publisher websites, the British Library and the Christian Medical College library in India. We excluded papers reporting a small number of cases (n < 10), no information about the number of S. Typhi or Paratyphi infections, no laboratory confirmation of infection (based on culture or serology), no distinction between *Salmonella* serovars, vaccine trials (unless cluster-randomized with a control arm), unknown geographical areas or outside India, or a review of the literature only. If the typhoid burden was reported multiple times for the same region, study population and time period, the study with the longest follow up time was selected.

Two reviewers (CVA and NCG) independently extracted data from the included studies and entered these into independent Excel databases. Disagreements between the two databases were resolved by consensus among all authors. Year of publication, study design, setting (hospital or community based study), study location, inclusion and exclusion criteria for study participants, start and end date of recruitment, type of samples, laboratory tests, whether the study was an outbreak report, number of participants, number of cases, age distribution of cases and sex of cases were collected. Longitude and latitude information of the study location was obtained from the US National Geospatial Intelligence Agency [22].

The outcome measures of interest were the prevalence of *S*. Typhi or Paratyphi among patients tested for infection in hospital settings or the incidence of typhoid and paratyphoid fever recorded in community studies. We did not publish our study protocol prior to completing the systematic review.

### Statistical analysis

We calculated the proportion of patients with laboratory confirmed typhoid or paratyphoid fever together with Wald 95% confidence intervals calculated on a logit scale for each study reporting data from hospitals [23]. Pooled estimates of prevalence were obtained by combining studies in a binomial regression model with a normally distributed random effect on the intercept. Heterogeneity between the studies was assessed using a likelihood ratio test (LRT) comparing a saturated mixed-effects model (with dummy variables for the random-effects) with a fixed-effects only model. A stratified analysis was performed due to anticipated heterogeneity between studies, based on the characteristics of the patients included in the studies (either fever described in the publication as suspected typhoid fever or fever where clinical suspicion of typhoid is either not present or not reported (hereafter just termed ‘fever’)). Independent variables potentially associated with the prevalence of typhoid or paratyphoid were included as fixed-effects in univariate and multivariate binomial meta-regression models.

The incidence of typhoid and paratyphoid was calculated per 100,000 person-years of observation for prospective community-based studies and Wald 95% confidence intervals calculated on a log scale. Pooled estimates of incidence were obtained by combining studies in a Poisson regression model with a normally distributed random effect on the intercept and heterogeneity assessed as above. Independent variables potentially associated with the incidence of typhoid or paratyphoid were included as fixed effects in univariate and multivariate Poisson meta-regression models.

Analyses were all performed in the R statistical programming language using the ‘metafor’ package [24,25].

## Results

### Characteristics of included studies

**Fig 1:**
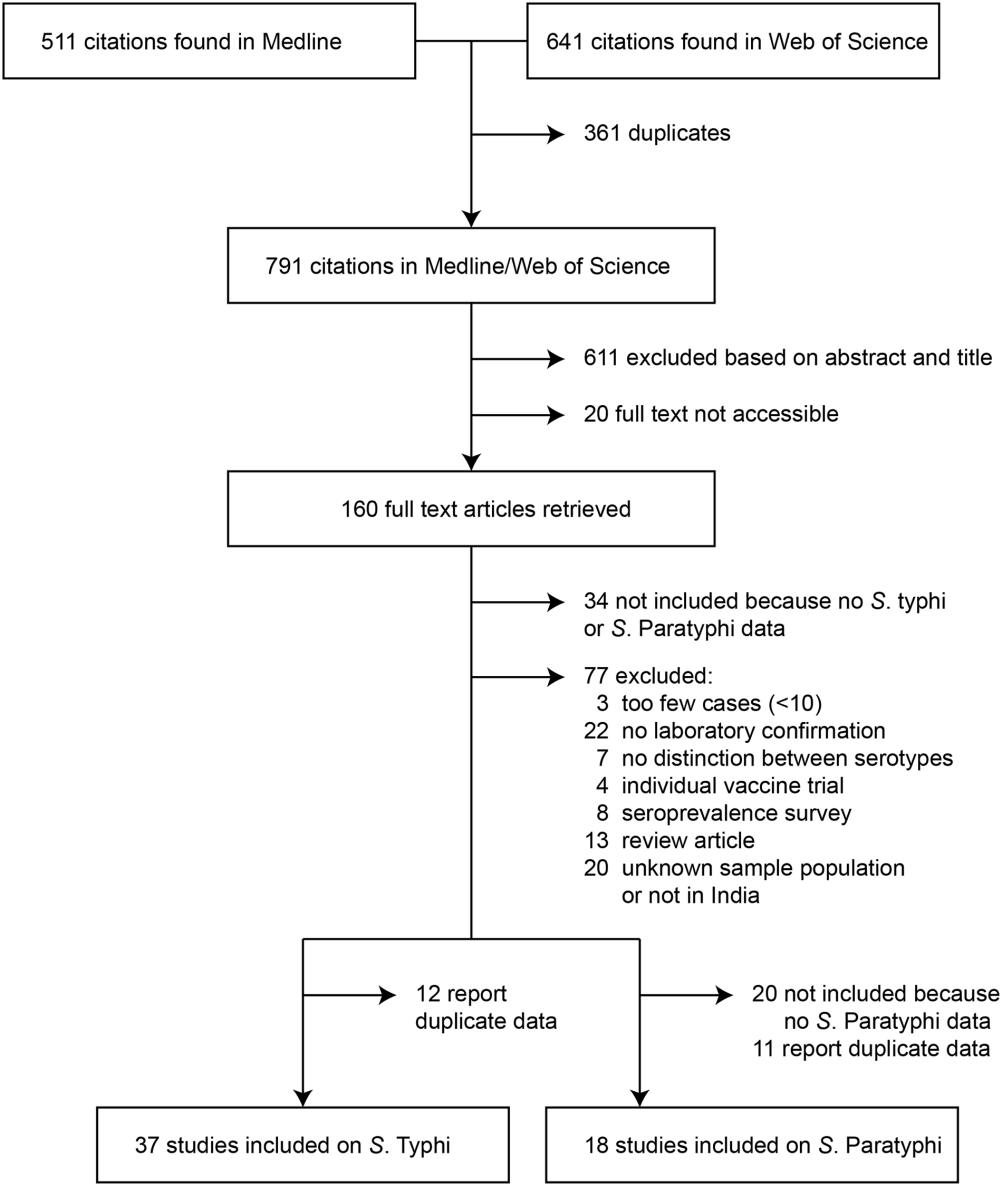
Flow diagram showing the number of articles identified in the systematic review on typhoid and paratyphoid in India.

The search strategy initially yielded 1,152 records of which 361 were duplicates (Fig 1). Six hundred and eleven records were excluded after screening the title and abstract. Full text copies were retrieved for 160 of 180 potential relevant records. After excluding non-eligible articles and duplicates, we included 37 studies that reported on typhoid and 18 that reported on paratyphoid. The characteristics of the included studies are given as Table A in the S1 Appendix.

Three studies of typhoid were community cohorts while the other 34 were conducted in hospitals, with all but 3 conducted in urban settings. Among the studies conducted in hospitals, 13 included participants with fever and 21 with suspected typhoid fever while all the community based studies included participants with fever. Thirty of the hospital studies and both the community studies reported culture confirmed typhoid, while four studies reported either a combination of culture and serology or serology alone. Studies reporting typhoid based on serology were included only if the serologic diagnostic criteria was clearly described.

All of the 18 studies reporting the prevalence of paratyphoid included *S*. Paratyphi A, and one described both *S*. Paratyphi A and B. Incidence of paratyphoid was described in two community cohort studies that reported from the same location (Kolkata).

### Hospital-based studies

**Fig 2:**
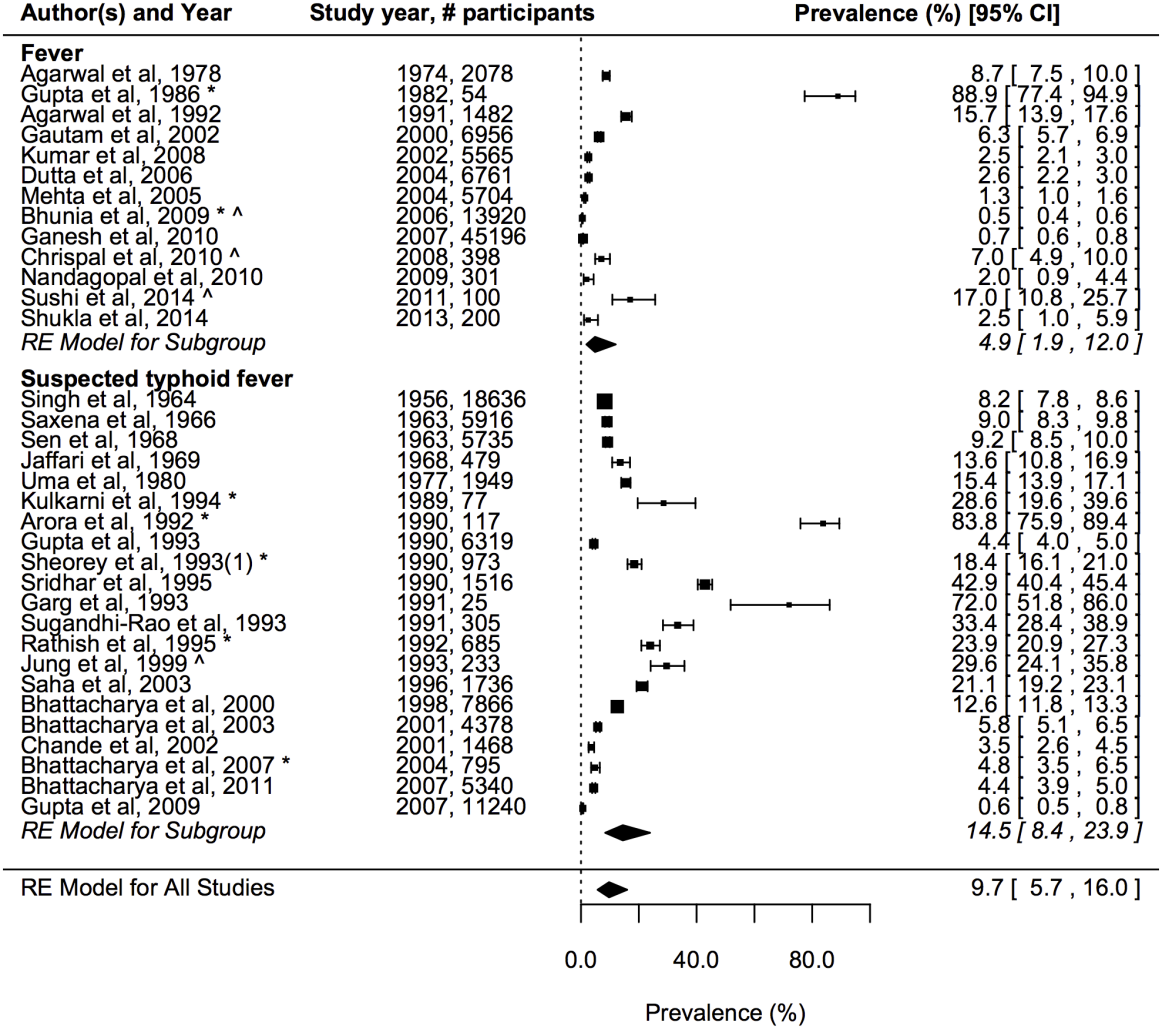
Prevalence of laboratory confirmed typhoid among patients with fever or suspected typhoid fever ordered by study year. Error bars indicate 95% confidence intervals, which are also given in square brackets for each study. Diamonds show the pooled estimates by patient group and overall together with 95% confidence intervals based on the fit of the random effects (RE) binomial (meta-) regression model. *indicates studies carried out during an outbreak of typhoid fever. ^indicates studies that used serology (alone or in addition to culture) to test for typhoid fever.

The estimated prevalence of *S*. Typhi detected through culture or serology across all hospital-based studies in the random effects model was 9.7% (95% confidence interval (CI): 5.7–16.0%) (Fig 2). There was significant heterogeneity in prevalence among studies (LRT p<0.001). Prevalence was higher among participants with suspected typhoid fever (estimated prevalence in separate random-effects model was 14.5%, 95% CI: 8.4–23.9%) compared with fever (estimated prevalence 4.9%, 95% CI: 1.9–12%). This was confirmed in the univariate mixed-effects, meta-regression model (Odds Ratio (OR) of laboratory confirmation for suspected typhoid fever compared with fever was 3.34, 95% CI: 1.11–10.1; p = 0.032) (Table 1). In the same analysis, study year (or midpoint for multiannual studies) was significantly associated with the odds of laboratory confirmation of typhoid. The OR was 0.95 (95% CI: 0.92–0.99) for each unit increment in the study year, although this decline is apparent in the forest plot only for studies from 1991 onwards (Fig 2). Typhoid was also more likely to be confirmed for studies that reported during an outbreak, although this was only of borderline significance in the univariate analysis (OR 3.66, 95% CI: 0.95–14.1; p = 0.060). Other study characteristics, including location (urban vs. rural, latitude) and type of laboratory assay (culture, serology or both) were not significantly associated with the odds of confirmation of typhoid. In the multivariate meta-regression model including all covariates, the year of the study and whether it reported during an outbreak were significantly associated with the odds of laboratory confirmation of typhoid (Table 2). The duration of fever among patients eligible for testing and their age distribution were available in only 9 of the 37 including studies and therefore subgroup and meta-regression analysis based on these variables were not carried out.

**Table 1:** Meta-regression of variables associated with the proportion of individuals who are confirmed to have typhoid fever based on a univariate and multivariate model.

Variable	Univariate	P-value	Multivariate	P-value
	Odds Ratio (95% CI)		Odds Ratio (95% CI)	
case definition				
- fever	-	-	-	-
- suspected typhoid fever	3.34 (1.11, 10.06)	*0*.*032*	1.53 (0.50, 4.66)	0.457
Outbreak (yes vs no)				
- no	-	-	-	-
- yes	3.66 (0.95, 14.10)	0.060	3.95 (1.19, 13.13)	*0*.*025*
Setting				
- rural	-	-	-	-
- urban	0.65 (0.09, 4.88)	0.673	0.92 (0.15, 5.69)	0.928
latitude (10^−6^ degrees)	0.76 (0.31, 1.84)	0.538	0.70 (0.30, 1.62)	0.402
Year of study (midpoint)	0.95 (0.92, 0.99)	*0*.*011*	0.96 (0.92, 0.99)	*0*.*022*
Year of study (midpoint) by decade:				
- 1950–1959	ref.		ref.	
- 1960–1969	1.3 (0.14, 12.05)		1.15 (0.14, 9.47)	
- 1970–1979	1.47 (0.14, 15.65)		1.13 (0.11, 12.14)	
- 1980–1989	18.94 (1.71, 209)		20.94 (1.4, 312)	
- 1990–1999	4.38 (0.58, 32.88)		3.28 (0.46, 23.62)	
- 2000–2009	0.27 (0.04, 1.99)		0.25 (0.03, 1.85)	
- 2010–2015	0.82 (0.07, 9.13)		1.07 (0.08, 14.23)	
Laboratory assay				
- culture	-	-	-	-
- culture or serology	1.57 (0.14, 16.93)	0.712	2.66 (0.33, 21.26)	0.357
- serology only	0.26 (0.02, 2.88)	0.275	0.40 (0.04, 3.74)	0.420

**Table 2:** Meta-regression of variables associated with the proportion of individuals who are confirmed to have paratyphoid fever based on a univariate and multivariate model.

Variable	Univariate	P-value	Multivariate	P-value
	Odds Ratio (95% CI)		Odds Ratio (95% CI)	
Case definition				
- fever	-	-	-	-
- suspected typhoid fever	0.73 (0.19, 2.77)	0.647	0.56 (0.17, 1.86)	0.344
Outbreak (yes vs no)				
- no	-	-	-	-
- yes	2.57 (0.43, 15.38)	0.302	4.16 (0.91, 19.04)	0.067
Setting				
- rural	-	-	-	-
- urban	0.09 (0.01, 0.73)	*0*.*025*	0.06 (0.01, 0.45)	*0*.*007*
latitude (10^−6^ degrees)	0.52 (0.23, 1.16)	0.111	0.96 (0.40, 2.32)	0.929
Year of study (midpoint)	1.01 (0.98, 1.05)	0.505	1.00 (0.97, 1.03)	0.881
Laboratory assay				
- culture	-	-	-	-
- culture or serology	1.03 (0.08, 13.83)	0.981	0.90 (0.07, 11.94)	0.936
- serology only	4.25 (0.32, 57.31)	0.276	3.69 (0.27, 49.56)	0.325

**Fig 3:**
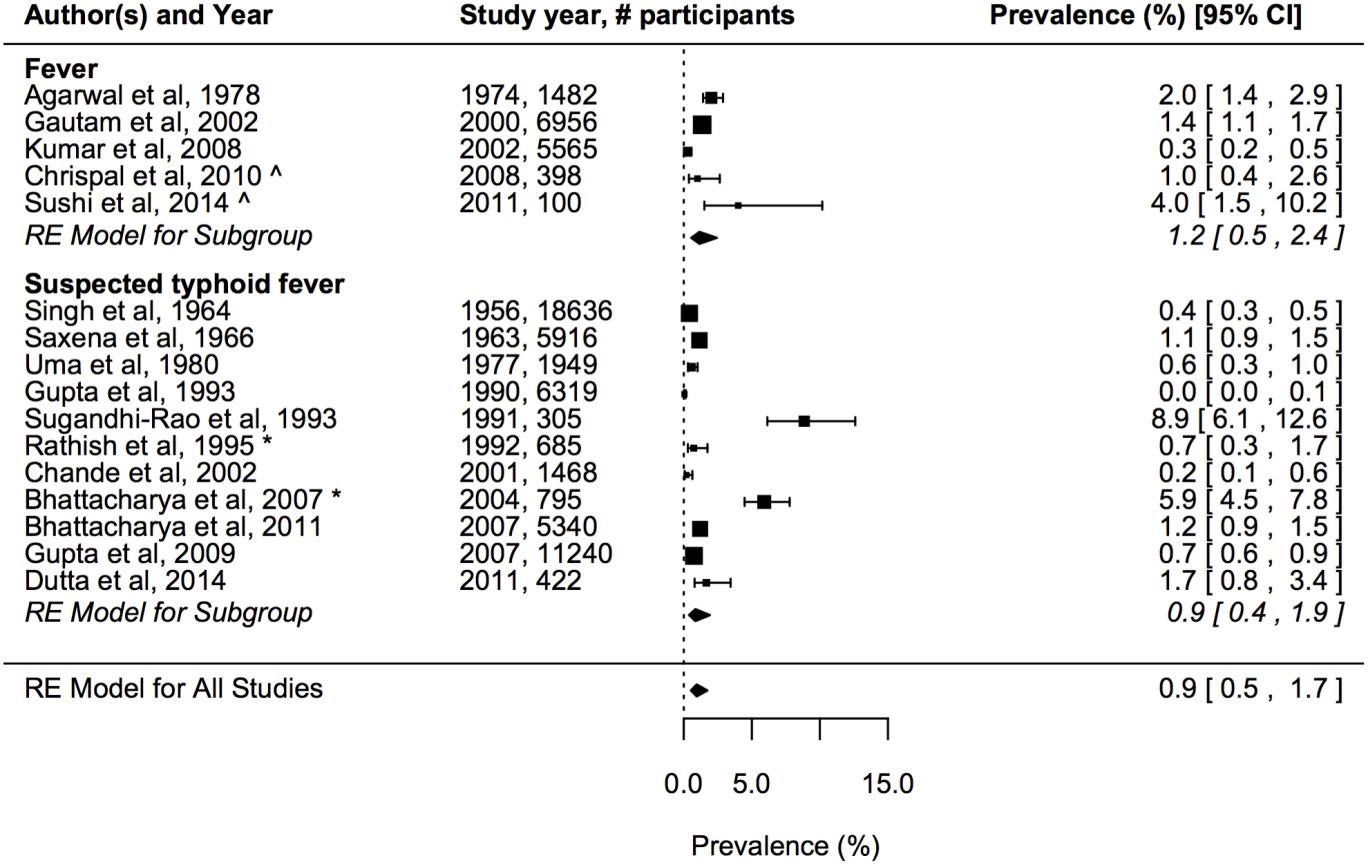
Prevalence of laboratory confirmed paratyphoid among patients with fever or suspected typhoid fever ordered by study year. Details as for Fig 2.

The estimated prevalence of laboratory confirmed paratyphoid across the hospital-based studies in the random effects model was 0.9% (95% CI: 0.5–1.7%) (Fig 3). There was significant heterogeneity among studies (LRT p<0.001). Prevalence was not significantly different according to whether studies included patients with fever or suspected typhoid fever. In the univariate and multivariate meta-regression models only location (urban vs. rural) was significantly associated with the prevalence of paratyphoid, although this was driven by a single rural study with high prevalence [26] (Table 2). In the multivariate meta-regression model, reporting during a typhoid outbreak was associated with an increased odds of laboratory confirmed paratyphoid of borderline statistical significance (OR 4.16, 95% CI: 0.91–19.0; p = 0.067).

Funnel plots of typhoid and paratyphoid prevalence against study size were strongly suggestive of publication bias, such that studies with high prevalence were more likely to be published (Figs A and B in S1 Appendix).

### Community cohort studies of incidence

**Fig 4:**
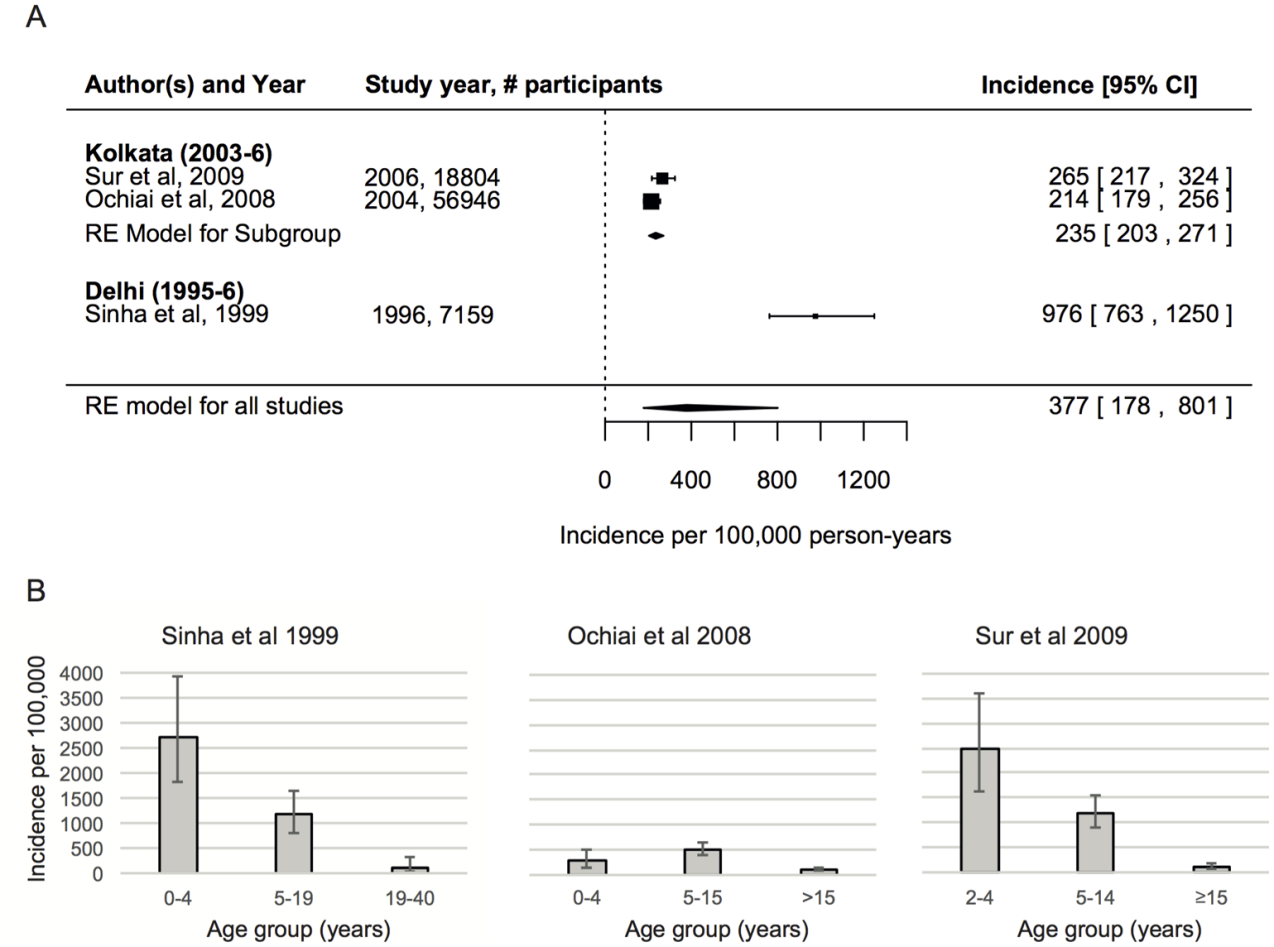
Incidence of typhoid fever based on community cohort studies. A) Incidence by study and pooled estimates (diamonds) are shown based on the fit of the random effect (RE) Poisson (meta-) regression model. The error bars and horizontal extent of the diamonds correspond with the 95% confidence intervals, which are also given in square brackets. B) Incidence by age-group for each study. Note differences in the definitions of the age-categories. We used the number of people enumerated at baseline to estimate the number of individuals at risk for the Kolkata 2004 estimate, since person-years of observation was not reported in this study [29].

The incidence of laboratory confirmed typhoid fever varied between the two locations where community cohort studies were carried out, with a more than four times higher incidence in Kalkaji (Delhi) of 976 per 100,000 person-years (95% CI: 736–1250) compared with Kolkata (pooled estimate 235, 95% CI: 203–271) (Fig 4A). Although the former study reported for individuals aged 0–40 years and the latter for all ages (under 2s were excluded in [27]), this does not explain this large difference in incidence, since individuals over 40 years old made up only 24% of the population in 2000 [28]. The pooled incidence across all studies was 377 (95% CI: 178–801) per 100,000 person-years although with significant heterogeneity among studies (LRT p<0.001). It was difficult to compare the age-distribution of typhoid incidence between studies because of differences in reporting of age-categories, although incidence was typically highest in the 2–4 year age-group (Fig 4B).

The incidence of paratyphoid was only reported for two studies in Kolkata that met our inclusion criteria, which gave a pooled estimate of incidence of 105 per 100,000 person years (95% CI: 74–148) for all ages (although [27] only reported from 2 years of age).

Note that the incidence of typhoid and paratyphoid in 2004 in Kolkata was estimated using number of individuals in the relevant study area and age-group at baseline because the number of person-years of observation was not reported [5,29].

## Discussion

There have, surprisingly, been very few epidemiological investigations of the incidence of typhoid in India. The three community cohort studies that we identified in Kolkata and Delhi, the last of which reported data nearly a decade back, suggest a variable incidence of typhoid both over time as well as across regions. The variable burden of typhoid was also apparent in the meta-analysis of hospital-based studies, which showed significant heterogeneity in the reported prevalence of laboratory confirmed typhoid among patients with fever or suspected typhoid fever.

In the meta-regression of hospital studies, testing of patients with suspected typhoid fever or during a typhoid outbreak was more likely to lead to laboratory confirmation of typhoid fever. The meta-regression also revealed a significant decline in laboratory confirmed typhoid among patients with fever or suspected typhoid fever over time, apparent since the early 1990s (Fig 2). The odds of detecting typhoid decreased by approximately 5% each year and this remained significant in the multivariate model accounting for differences in study location, laboratory assay, case definition and whether reporting was during an outbreak. Grouping the studies by decade shows that this significant decline is largely the result of the high prevalence of typhoid in hospital studies during 1980–2000 compared with more recent studies (Table 1). The cause of the high rate of typhoid isolation at this time is not clear. Moreover, inference of a trend from hospital-based studies in different locations and with variable health-seeking behaviours must be tentative. In particular, it is possible that increased use of effective antibiotics before blood collection could have contributed to this decline. The prevalence of typhoid fever was not significantly associated with any other covariates, including study location (latitude, urban vs. rural), although the number of studies in rural areas was small, limiting the power of this analysis.

The incidence of paratyphoid was reported in only two studies that met our inclusion criteria, both in Kolkata. The estimated incidence of paratyphoid in this setting was 105 per 100,000 person years, which compared with 235 per 100,000 person years for typhoid. In Kalkaji (Delhi) the incidence of paratyphoid was not originally reported, although in a companion publication [30] the number of paratyphoid cases recorded during a slightly longer follow-up (18 months) compared with the original study (12 months) [31] was 31 compared with 98 for typhoid over the same period. These estimates suggest an incidence rate for paratyphoid in these settings that is about 30–50% of the rate estimated for typhoid. The lower incidence of paratyphoid was confirmed in the meta-regression of hospital-based studies, which found a prevalence for paratyphoid that was approximately 10-fold lower than for typhoid (estimated pooled prevalence of 0.9% vs. 10.7%). The significantly lower prevalence of paratyphoid among patients tested in hospital may also reflect the shorter duration of fever and more mild clinical characteristics of paratyphoid compared with typhoid [32]. Although significant heterogeneity in the prevalence of paratyphoid was identified among the hospital-based studies, this was not associated with case definition, laboratory assay, whether an outbreak was reported or any other study covariates, with the exception of study location. Prevalence was significantly higher in rural compared with urban locations, but this result was driven by a single study of paratyphoid in a rural area that had high prevalence.

Consistent across the community cohort studies was the finding of a high incidence of typhoid in children under five years of age, suggestive of a substantial burden in a group that would benefit from infant rather than school-based immunization. This is consistent with recent recommendations from the IAP on the creation of an immunisation slot at 9–12 months of age for typhoid vaccination [21]. Risk-factors that would allow targeting of infant immunization to high-risk groups were not identified in this systematic review and meta-analysis. Significant heterogeneity was observed among studies, but this is likely in part to reflect differences in patient inclusion criteria, laboratory methods and changes in antimicrobial use and resistance patterns. Vaccine introduction is likely to be more sustainable, equitable and to provide indirect herd effects when it is done through the universal immunisation programme.

The age-distribution of paratyphoid incidence was not reported, although the mean age in 2004 in Kolkata was reported as being significantly higher compared with typhoid (17.1 vs. 14.7 years)[29]. Paratyphoid vaccines are not yet available and currently licensed typhoid vaccines offer limited or no protective immunity against paratyphoid A and B, the predominant serotypes [33]. However, vaccines are in the development pipeline, including bivalent conjugate vaccines that could offer protection against both typhoid and paratyphoid.

There were limitations common to published studies that hampered our systematic review of the burden and risk factors for typhoid and paratyphoid fever in India. There have been only three community cohort studies of incidence, in just two locations and with highly variable findings. Although far more numerous, the hospital-based studies have a number of limitations. Firstly, hospital studies provide no information about incidence without a detailed understanding of local health-seeking behaviour—something missing from published studies. Secondly, they had varying inclusion criteria for patients, sample collection and laboratory methods, making interpretation of these data challenging. We focussed on laboratory confirmed typhoid or paratyphoid fever, mostly blood culture. However, blood culture has a poor sensitivity of about 50% and is strongly influenced by the quantity of blood, prior administration of antibiotics and culture techniques including quality of media [34]. We excluded cross-sectional community studies using serology, since these were likely to be highly non-specific for typhoid. Thirdly, detailed data on the inclusion criteria for patients including the duration of fever and age-distribution were usually missing, limiting the number of covariates we could include in the meta-analysis. In some studies, a failure to clearly define inclusion criteria along with ambiguous reporting forced us to exclude them because the denominator population was unclear. Fourthly, most studies did not report clinical outcomes and therefore we were unable to evaluate trends in severe disease or mortality. Finally, there was evidence from the funnel plots for publication bias, such that studies finding a high burden of typhoid or paratyphoid were more likely to be reported and published. Therefore, while there appears to be a declining trend in typhoid isolation in hospitals, drawing inference about the underlying burden of disease from hospital based data needs to be approached cautiously.

The limitations of the community cohort and hospital-based studies make it difficult to estimate the total burden of typhoid and paratyphoid in India. Extrapolating the estimates of typhoid incidence from Kolkata and Delhi to the rest of India is clearly problematic. A naïve approach applying the pooled estimate of the incidence rate to the 2011 census population of 1.2 billion would give an estimated annual incidence of 4.6 million cases. This could be revised upwards by approximately twofold based on the poor sensitivity of culture-based confirmation of typhoid [2]. However, the community cohort studies were deliberately planned in densely populated urban areas with poor sanitation, likely to have a high incidence of typhoid. Correcting the national estimate for access to improved water following the approach used for regional estimates by Mogasale et al. 2014 [2] would give 2.1 million cases annually, or approximately 3.4 million correcting for imperfect culture sensitivity. Correction for other risk factors, such as population density, would likely reduce this estimate further.

Strengthening surveillance across geographically representative sentinel sites is key to better disease burden estimates. Inclusion of other data sources such as large healthcare facilities, and the National Disease Surveillance Project is likely to further understanding of disease burden.

Well defined surveillance criteria combined with standardized laboratory methods will greatly enhance comparability of estimates from diverse sites. Since blood cultures are highly dependent on volume of inoculum, prior antibiotic exposure and laboratory methods, a combination of conventional, molecular and serologic diagnostics modalities would probably be optimal. Information about time trends and antimicrobial resistance patterns that arise from such a systematic surveillance will enhance our understanding of typhoid and paratyphoid in India and strengthen public health decision making.

## Supporting Information

S1 Appendix Search terms and summary of included studies. (DOCX)

S1 File PRISMA checklist. (DOC)

S1 Fig Funnel plot showing the prevalence of laboratory confirmed typhoid among patients with fever or suspected typhoid fever ordered by number of patients tested. (TIF)

S2 Fig Funnel plot showing the prevalence of laboratory confirmed paratyphoid among patients with fever or suspected typhoid fever ordered by number of patients tested. (TIF)

S3 Fig Map of the number of studies included in the systematic review by location. (TIF)

S1 Data Data for included studies on typhoid. (TXT)

S2 Data Data for included studies on paratyphoid. (TXT)

## References

[1] Buckle GC, Walker CL, Black RE (2012) Typhoid fever and paratyphoid fever: Systematic review to estimate global morbidity and mortality for 2010. J Glob Health 2: 010401 23198130 10.7189/jogh.02.010401 PMC3484760

[2] Mogasale V, Maskery B, Ochiai RL, Lee JS, Mogasale VV, et al (2014) Burden of typhoid fever in low-income and middle-income countries: a systematic, literature-based update with risk-factor adjustment. Lancet Global Health 2: E570–E580. 10.1016/S2214-109X(14)70301-8 25304633

[3] Wain J, Hendriksen RS, Mikoleit ML, Keddy KH, Ochiai RL (2014) Typhoid fever. The Lancet. 10.1016/S0140-6736(13)62708-7 PMC11567078 25458731

[4] Crump JA, Mintz ED (2010) Global Trends in Typhoid and Paratyphoid Fever. Clinical Infectious Diseases 50: 241–246. 10.1086/649541 20014951 PMC2798017

[5] Ochiai RL, Acosta CJ, Danovaro-Holliday MC, Baiqing D, Lhattacharya SK, et al (2008) A study of typhoid fever in five Asian countries: disease burden and implications for controls. Bulletin of the World Health Organization 86: 260–268. 18438514 10.2471/BLT.06.039818 PMC2647431

[6] Ochiai RL, Wang XY, von Seidlein L, Yang J, Bhutta ZA, et al (2005) Salmonella paratyphi A rates, Asia. Emerging Infectious Diseases 11: 1764–1766. 16318734 10.3201/eid1111.050168 PMC3367370

[7] Sur D, Ali M, von Seidlein L, Manna B, Deen JL, et al (2007) Comparisons of predictors for typhoid and paratyphoid fever in Kolkata, India. BMC Public Health 7: 289 17935611 10.1186/1471-2458-7-289 PMC2099435

[8] Bhunia R, Hutin Y, Ramakrishnan R, Pal N, Sen T, et al (2009) A typhoid fever outbreak in a slum of South Dumdum municipality, West Bengal, India, 2007: Evidence for foodborne and waterborne transmission. Bmc Public Health 9. 10.1186/1471-2458-9-115 PMC2683821 19397806

[9] Ames WR, Robins M (1943) Age and Sex as Factors in the Development of the Typhoid Carrier State, and a Method for Estimating Carrier Prevalence. American journal of public health and the nation's health 33: 221–230. 18015749 10.2105/ajph.33.3.221 PMC1527221

[10] Pitzer VE, Bowles CC, Baker S, Kang G, Balaji V, et al (2014) Predicting the Impact of Vaccination on the Transmission Dynamics of Typhoid in South Asia: A Mathematical Modeling Study. PLoS Negl Trop Dis 8: e2642. 24416466 10.1371/journal.pntd.0002642 PMC3886927

[11] Anwar E, Goldberg E, Fraser A, Acosta CJ, Paul M, et al (2014) Vaccines for preventing typhoid fever. Cochrane Database of Systematic Reviews 96. 10.1002/14651858.CD001261.pub3 24385413

[12] Levine MM, Black RE, Ferreccio C, Germanier R (1987) Large-scale field trial of Ty21a live oral typhoid vaccine in enteric-coated capsule formulation. Lancet 1: 1049–1052. 2883393 10.1016/s0140-6736(87)90480-6

[13] Levine MM, Ferreccio C, Abrego P, San Martin O, Ortiz E, et al (1999) Duration of efficacy of Ty21a, attenuated Salmonella typhi live oral vaccine. Vaccine 17: S22–S27. 10506405 10.1016/s0264-410x(99)00231-5

[14] Acharya IL, Lowe CU, Thapa R, Gurubacharya VL, Shrestha MB, et al (1987) Prevention of typhoid-fever in Nepal with the Vi capsular polysaccharide of *Salmonella Typhi*—a preliminary-report. N Engl J Med 317: 1101–1104. 3657877 10.1056/NEJM198710293171801

[15] Klugman KP, Koornhof HJ, Robbins JB, LeCam NN (1996) Immunogenicity, efficacy and serological correlate of protection of Salmonella typhi Vi capsular polysaccharide vaccine three years after immunization. Vaccine 14: 435–438. 8735556 10.1016/0264-410x(95)00186-5

[16] Lin FYC, Ho VA, Khiem HB, Trach DD, Bay PV, et al (2001) The efficacy of a Salmonella typhi Vi conjugate vaccine in two-to-five-year-old children. New England Journal of Medicine 344: 1263–1269. 11320385 10.1056/NEJM200104263441701

[17] Lanh MN, Bay PV, Ho VA, Thanh TC, Lin FYC, et al (2003) Persistent efficacy of Vi conjugate vaccine against typhoid fever in young children. New England Journal of Medicine 349: 1390–1391. 14523155 10.1056/NEJM200310023491423

[18] Szu SC (2013) Development of Vi conjugate—a new generation of typhoid vaccine. Expert Rev Vaccines 12: 1273–1286. 10.1586/14760584.2013.845529 24156285

[19] Thiem VD, Lin FY, Canh do G, Son NH, Anh DD, et al (2011) The Vi conjugate typhoid vaccine is safe, elicits protective levels of IgG anti-Vi, and is compatible with routine infant vaccines. Clin Vaccine Immunol 18: 730–735. 10.1128/CVI.00532-10 21411598 PMC3122535

[20] World Health Organisation (2008) Typhoid vaccines: WHO position paper. Wkly Epidemiol Rec 83: 49–60. 18260212

[21] Vashishtha VM, Choudhury P, Kalra A, Bose A, Thacker N, et al (2014) Indian Academy of Pediatrics (IAP) Recommended Immunization Schedule for Children Aged 0 through 18-years India, 2014 and Updates on Immunization. Indian Pediatrics 51: 785–800. 25362009 10.1007/s13312-014-0504-y

[22] Clarke H, Bonin RP, Orser BA, Englesakis M, Wijeysundera DN, et al (2012) The prevention of chronic postsurgical pain using gabapentin and pregabalin: a combined systematic review and meta-analysis. Anesth Analg 115: 428–442. 22415535 10.1213/ANE.0b013e318249d36e

[23] Newcombe RG (2001) Logit Confidence Intervals and the Inverse Sinh Transformation. The American Statistician 55: 200–202.

[24] R Development Core Team (2014) R: A language and environment for statistical computing. Vienna, Austria: R Foundation for Statistical Computing.

[25] Viechtbauer Wolfgang (2010) Conducting meta-analyses in R with metafor package. Journal of Statistical Software 36: 1–48.

[26] Rao PS, Rajashekar V, Varghese GK, Shivananda PG (1993) Emergence of multidrug-resistant Salmonella typhi in rural southern India. Am J Trop Med Hyg 48: 108–111. 8427378 10.4269/ajtmh.1993.48.108

[27] Sur D, Ochiai RL, Bhattacharya SK, Ganguly NK, Ali M, et al (2009) A cluster-randomized effectiveness trial of Vi typhoid vaccine in India. N Engl J Med 361: 335–344. 10.1056/NEJMoa0807521 19625715

[28] (2015) World Population Prospects: The 2015 Revision. In: Department of Economic and Social Affairs PD, editor: United Nations.

[29] Sur D, Ali M, von Seidlein L, Manna B, Deen JL, et al (2007) Comparisons of predictors for typhoid and paratyphoid fever in Kolkata, India. BMC Public Health 7: 289 17935611 10.1186/1471-2458-7-289 PMC2099435

[30] Bahl R, Sinha A, Poulos C, Whittington D, Sazawal S, et al (2004) Costs of illness due to typhoid fever in an Indian urban slum community: implications for vaccination policy. J Health Popul Nutr 22: 304–310. 15609783

[31] Sinha A, Sazawal S, Kumar R, Sood S, Reddaiah VP, et al (1999) Typhoid fever in children aged less than 5 years. Lancet 354: 734–737. 10475185 10.1016/S0140-6736(98)09001-1

[32] Bhan MK, Bahl R, Bhatnagar S (2005) Typhoid and paratyphoid fever. Lancet 366: 749–762. 16125594 10.1016/S0140-6736(05)67181-4

[33] Levine MM, Ferreccio C, Black RE, Lagos R, San Martin O, et al (2007) Ty21a live oral typhoid vaccine and prevention of paratyphoid fever caused by Salmonella enterica Serovar Paratyphi B. Clin Infect Dis 45 Suppl 1: S24–28. 17582564 10.1086/518141

[34] Kundu R, Ganguly N, Ghosh TK, Yewale VN, Shah RC, et al (2006) IAP Task Force Report: diagnosis of enteric fever in children. Indian Pediatr 43: 875–883. 17079830

