# Supplementary material for: The Burden of Typhoid and Paratyphoid in India: Systematic Review and Meta-analysis
Source: PLoS Negl Trop Dis. 2016 Apr 15;10(4):e0004616. doi: 10.1371/journal.pntd.0004616 (PMC4833325; doi:10.1371/journal.pntd.0004616)

**Text**

Complete search term used in Web of Science and PubMed databases on 15 May 2015.

((("Typhoid Fever"[Mesh]) OR "Salmonella typhi"[Mesh] OR typhoid OR "typhoid fever" OR "enteric fever" OR "Salmonella typhi" OR "Salmonella enterica" OR paratyphoid OR paratyphi) AND (("India"[Mesh]) OR India OR "Tamil Nadu" OR Kolkata OR Karnataka OR Chandigarh OR Odisha OR Assam OR “Madhya Pradesh” OR Kerala OR Chhattisgarh OR Nagaland OR Rajasthan OR “Himachal Pradesh” OR “West Bengal” OR “Jammu and Kashmir” OR Sikkim OR Gujarat OR Tripura OR Maharashtra OR “Andhra Pradesh” OR “Andaman and Nicobar” OR Goa OR Delhi OR Uttarakhand OR Manipur OR “Uttar Pradesh” OR Meghalaya OR Lakshadweep OR Puducherry OR Punjab OR Bihar OR Haryana OR “Daman and Diu” OR Mizoram OR Jharkhand) AND (epidemiology OR incidence OR prevalence OR mortality OR burden OR (((("Epidemiology"[Mesh]) OR "Incidence"[Mesh]) OR "Prevalence"[Mesh]) OR "Mortality"[Mesh]) OR "Cost of Illness"[Mesh]))

**Table 1: Summary of characteristics of included studies**

| **Studies** | **State** | **City** | **Setting** | **Study period** | **Number of participants** | **Included for typhoid** | **Included for Paratyphoid** | **Blood culture** | **Serology** | **All ages** | **Age data available** |
| --- | --- | --- | --- | --- | --- | --- | --- | --- | --- | --- | --- |
| Agarwal et al, 1978 | Uttar Pradesh | Lucknow | Hospital | 1973 - 1975 | 2078 (1482)* | Yes | Yes | + |  | Yes | No |
| Agarwal et al, 1992 | Maharashtra | Nagpur | Hospital | 1990 – 1991 | 1,482 | Yes | No | + |  | Yes | No |
| Arora et al, 1992 | West Bengal | Kolkata | Hospital | 1989 –1990 | 117 | Yes | No | + |  | No | No |
| Bhattacharya et al, 2000 | Odisha | Rourkela | Hospital | 1996 – 1998 | 7,866 | Yes | No | + |  | Yes | No |
| Bhattacharya et al, 2003 | Odisha | Rourkela | Hospital | 1999 – 2001 | 4,378 | Yes | No | + |  | Yes | No |
| Bhattacharya et al, 2007 | Odisha | Rourkela | Hospital | 2004 | 795 | Yes | Yes | + |  | Yes | No |
| Bhattacharya et al, 2011 | Odisha | Rourkela | Hospital | 2005 – 2008 | 5,340 | Yes | Yes | + |  | Yes | No |
| Bhunia et al, 2009 | West Bengal | Kolkata | Hospital | 2004 – 2007 | 13920 | Yes | No |  | + | Yes | No |
| Chande et al, 2002 | Maharashtra | Nagpur | Hospital | 2000 – 2001 | 1468 | Yes | Yes | + |  | Yes | No |
| Chrispal et al, 2010 | Tamil Nadu | Vellore | Hospital | 2007 – 2008 | 398 | Yes | Yes | + | + | No | No |
| Dutta et al, 2006 | West Bengal | Kolkata | Hospital | 2003 – 2004 | 6,761 | Yes | No | + |  | Yes | No |
| Dutta et al, 2014 | West Bengal | Kolkata | Hospital | 2009-2013 | 422 | No | Yes | + |  | No | No |
| Ganesh et al, 2010 | Tamil Nadu | Chennai | Hospital | 2005 – 2008 | 45,196 | Yes | No | + |  | No | Yes |
| Garg et al, 1993 | Delhi | Delhi | Hospital | 1990 – 1991 | 25 | Yes | No | + |  | No | No |
| Gautam et al, 2002 | Haryana | Rohtak | Hospital | 1997 – 2001 | 6,956 | Yes | Yes | + |  | Yes | No |
| Gupta et al, 1986 | Chandigarh | Chandigarh | Hospital | 1992 – 1982 | 54 | Yes | No | + |  | Yes | No |
| Gupta et al, 1993 | Punjab | Ludhiana | Hospital | 1990 – 1990 | 6,319 | Yes | Yes | + |  | Yes | No |
| Gupta et al, 2009 | Chandigarh | Chandigarh | Hospital | 2006 – 2007 | 11,240 | Yes | Yes | + |  | Yes | No |
| Jaffari et al, 1969 | Telangana | Hyderabad | Hospital | 1968 –1968 | 479 | Yes | No | + |  | No | No |

| **Studies** | **Indian state** | **City** | **Setting** | **Study period** | **Number of participants** | **Included for typhoid** | **Included for Paratyphoid** | **Blood culture** | **Serology** | **All ages** | **Age data available** |
| --- | --- | --- | --- | --- | --- | --- | --- | --- | --- | --- | --- |
| Jung et al, 1999 | Maharashtra | Wardha | Hospital | 1993 | 233 | Yes | No | + | + | No | No |
| Kulkarni et al, 1994 | Karnataka | Gulbarga | Hospital | 1989 – 1989 | 77 | Yes | No | + |  | No | Yes |
| Kumar et al, 2008 | Delhi | Delhi | Hospital | 1999-2005 | 5565 | Yes | Yes | + |  | Yes | No |
| Mehta et al, 2005 | Punjab | Chandigarh | Hospital | 2003-2004 | 5704 | Yes | No | + |  | Yes | No |
| Nandagopal et al, 2010 | Tamil Nadu | Vellore | Hospital | 2008-2009 | 301 | Yes | No | + |  | No | No |
| Ochiai, 2008 | West Bengal | Kolkata | Community | 2003-2004 | 56946 | Yes | No | + |  | Yes | Yes |
| Sugandhi-Rao et al, 1993 | Karnataka | Manipal | Hospital | 1990 – 1991 | 305 | Yes | Yes | + |  | Yes | No |
| Rathish et al, 1995 | Karnataka | Bangalore | Hospital | 1991 –1992 | 685 | Yes | Yes | + |  | No | No |
| Saha et al, 2003 | West Bengal | Kolkata | Hospital | 1990 - 2002 | 1,736 | Yes | No | + |  | Yes | Yes |
| Saxena et al, 1966 | Delhi | Delhi | Hospital | 1962 – 1964 | 5916 | Yes | Yes | + |  | No | No |
| Sen et al, 1968 | Delhi | Delhi | Hospital | 1962 – 1964 | 5735 | Yes | No | + |  | No | No |
| Sheorey et al, 1993 | Maharashtra | Mumbai | Hospital | 1988– 1990 | 973 | Yes | No | + |  | Yes | No |
| Shukla et al, 2014 | Uttar Pradesh | Barabaki | Hospital | 2012-2013 | 200 | Yes | No | + |  | Yes | No |
| Singh et al, 1964 | Delhi | Delhi | Hospital | 1950 - 1961 | 18,636 | Yes | Yes | + |  | Yes | No |
| Sinha et al, 1999 | Delhi | Kalkaji | Community | 1995 – 1996 | 8,172 | Yes | No | + |  | No | Yes |
| Sridhar et al, 1995 | Karnataka | Bangalore | Hospital | 1987 - 1992 | 1,516 | Yes | No | + |  | Yes | Yes |
| Sur et al, 2007 | West Bengal | Kolkata | Hospital | 2003-2004 | 127 | No | Yes | + |  | Yes | No |
| Sur et al, 2009 | West Bengal | Kolkata | Community | 2004 – 2006 | 18,804 | Yes | Yes | + |  | No | Yes |
| Sushi et al, 2014 | Tamil Nadu | Chennai | Hospital | 2011 – 2011 | 100 | Yes | Yes |  | + | Yes | No |
| Uma et al, 1980 | Tamil Nadu | Madurai | Hospital | 1976 – 1977 | 1,949 | Yes | Yes | + |  | Yes | No |

**Figure A** Funnel plot showing the prevalence of laboratory confirmed typhoid among patients with fever or suspected typhoid fever ordered by number of patients tested. Error bars indicate 95% confidence intervals, which are also given in square brackets for each study. Diamonds show the pooled estimates by patient group and overall together with 95% confidence intervals based on the fit of the random effects (RE) binomial (meta-) regression model. *indicates studies carried out during an outbreak of typhoid fever. ^indicates studies that used serology (alone or in addition to culture) to test for typhoid fever.


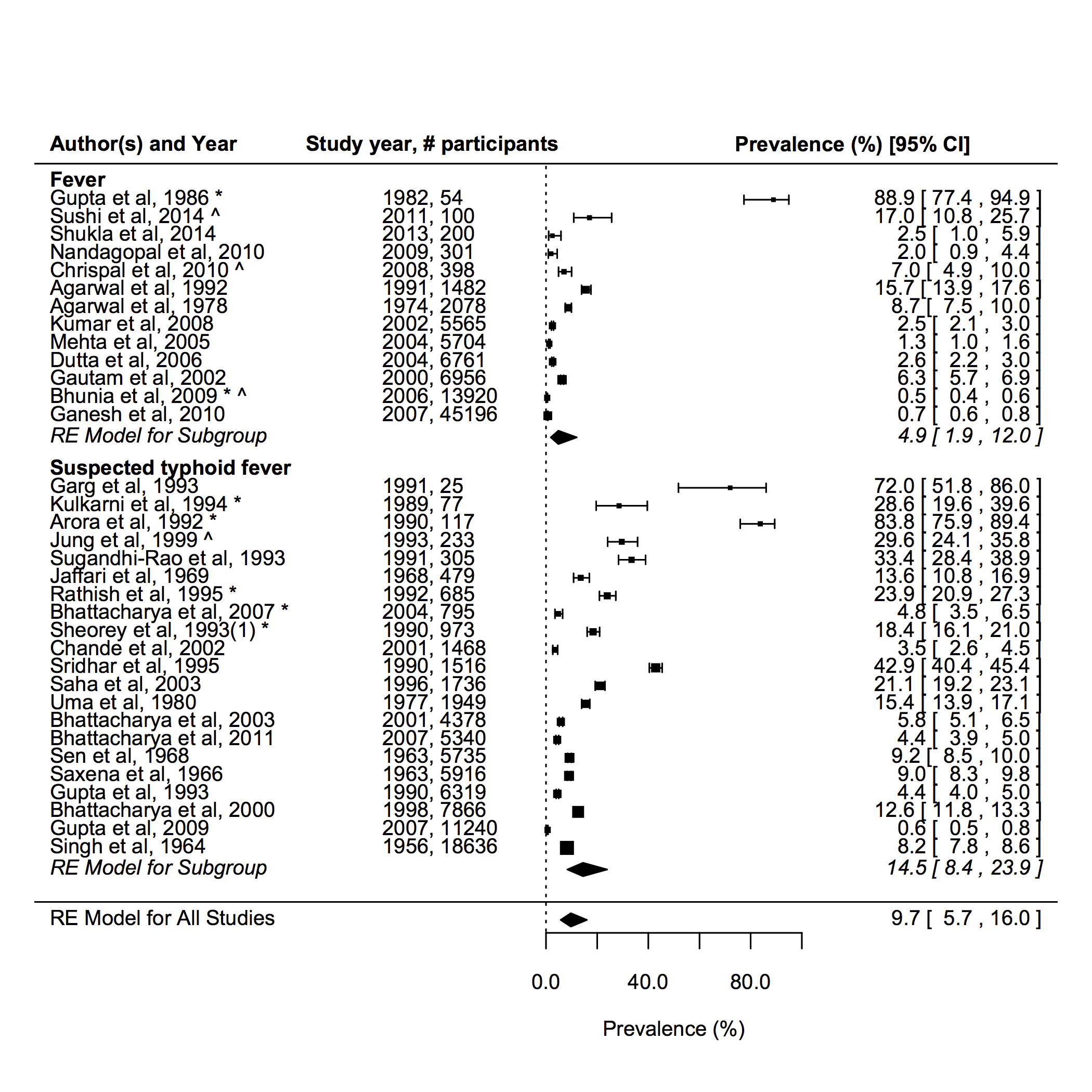


**Figure B** Funnel plot showing the prevalence of laboratory confirmed paratyphoid among patients with fever or suspected typhoid fever ordered by number of patients tested. Detailed legend as for Supplementary Figure A.


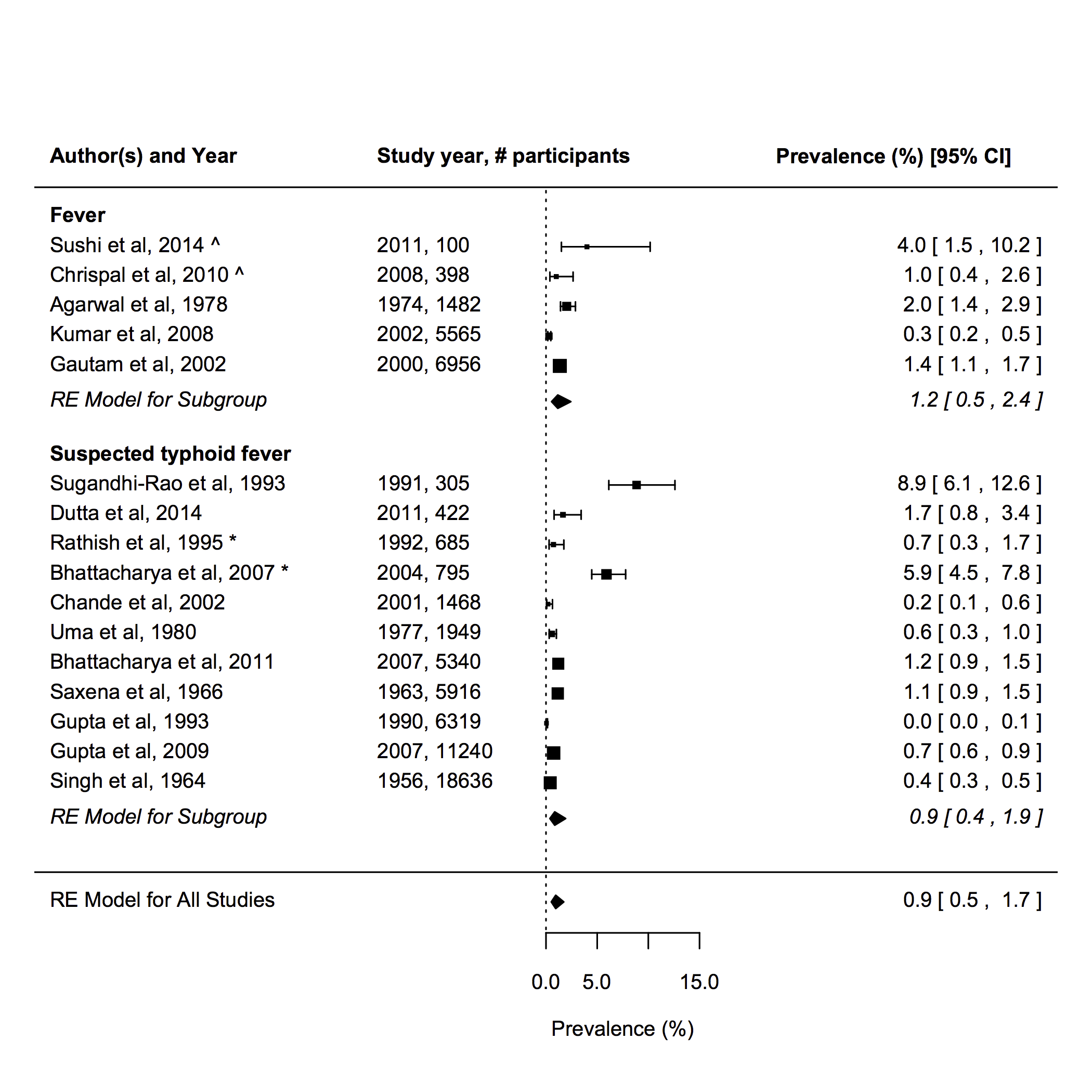

Supplement: S1 Appendix — (DOCX) [file pntd.0004616.s001.docx]
