# Supplementary figures and images for: The Burden of Typhoid and Paratyphoid in India: Systematic Review and Meta-analysis
Source: PLoS Negl Trop Dis. 2016 Apr 15;10(4):e0004616. doi: 10.1371/journal.pntd.0004616 (PMC4833325; doi:10.1371/journal.pntd.0004616)

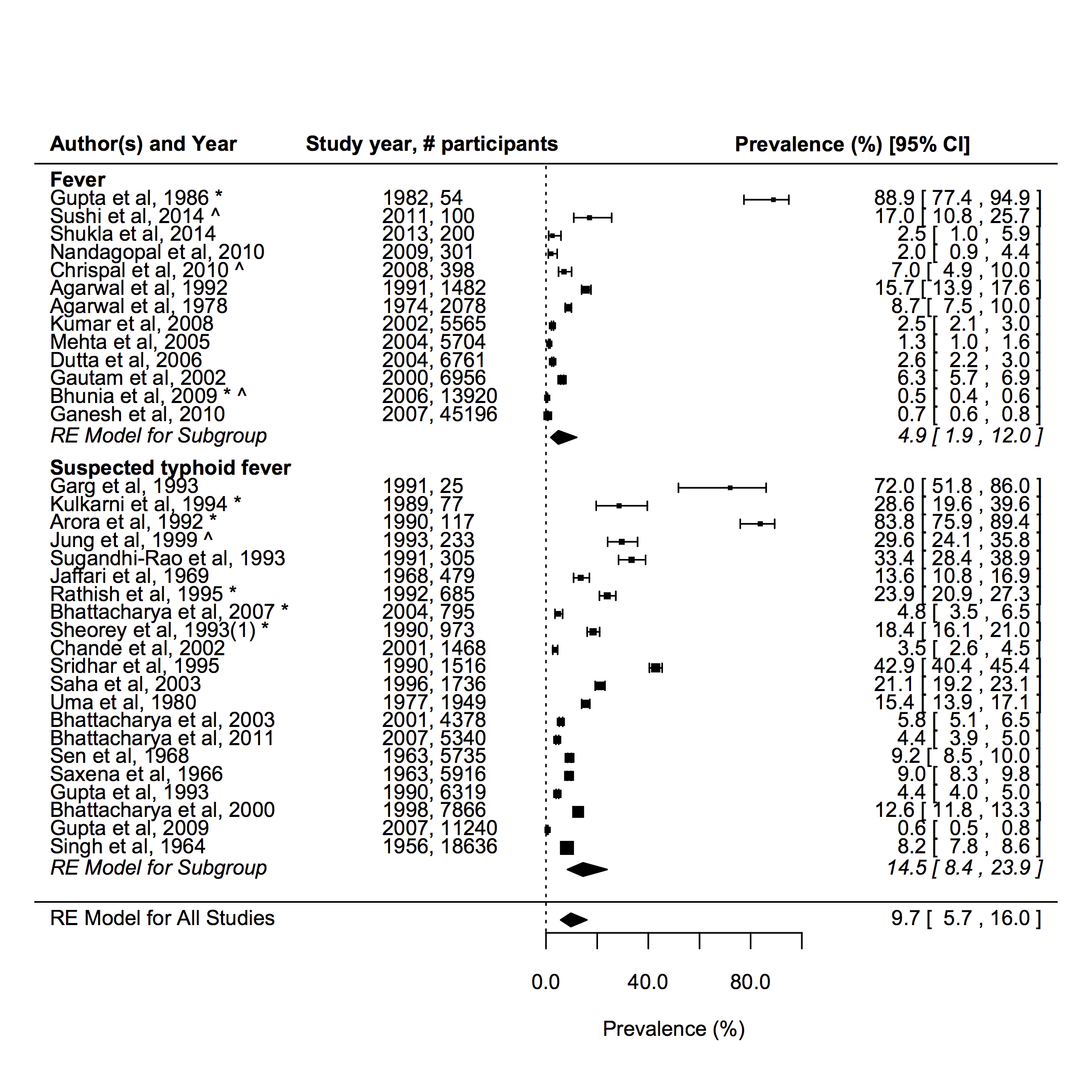

Supplement: S1 Fig — (TIF) [file pntd.0004616.s003.tif]

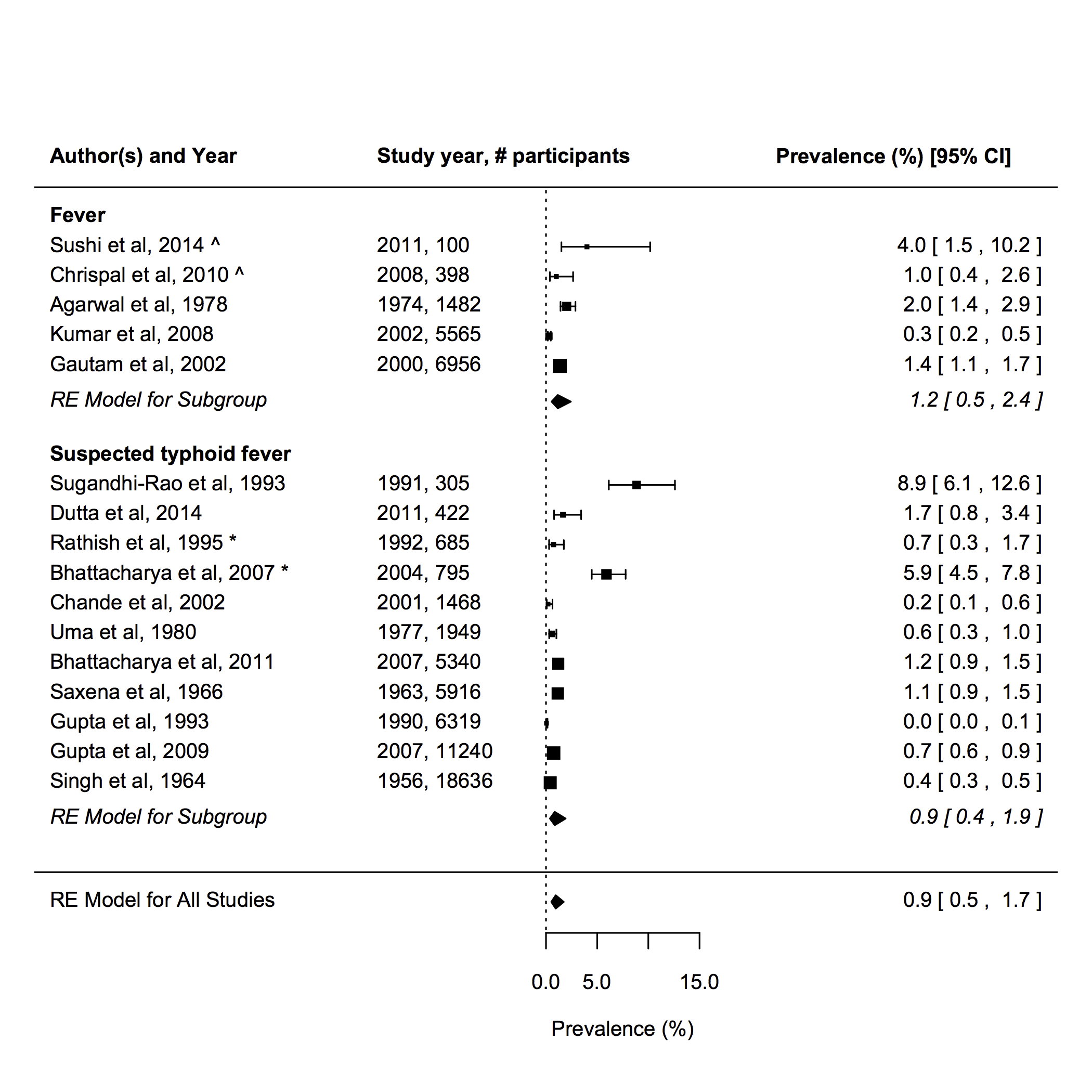

Supplement: S2 Fig — (TIF) [file pntd.0004616.s004.tif]

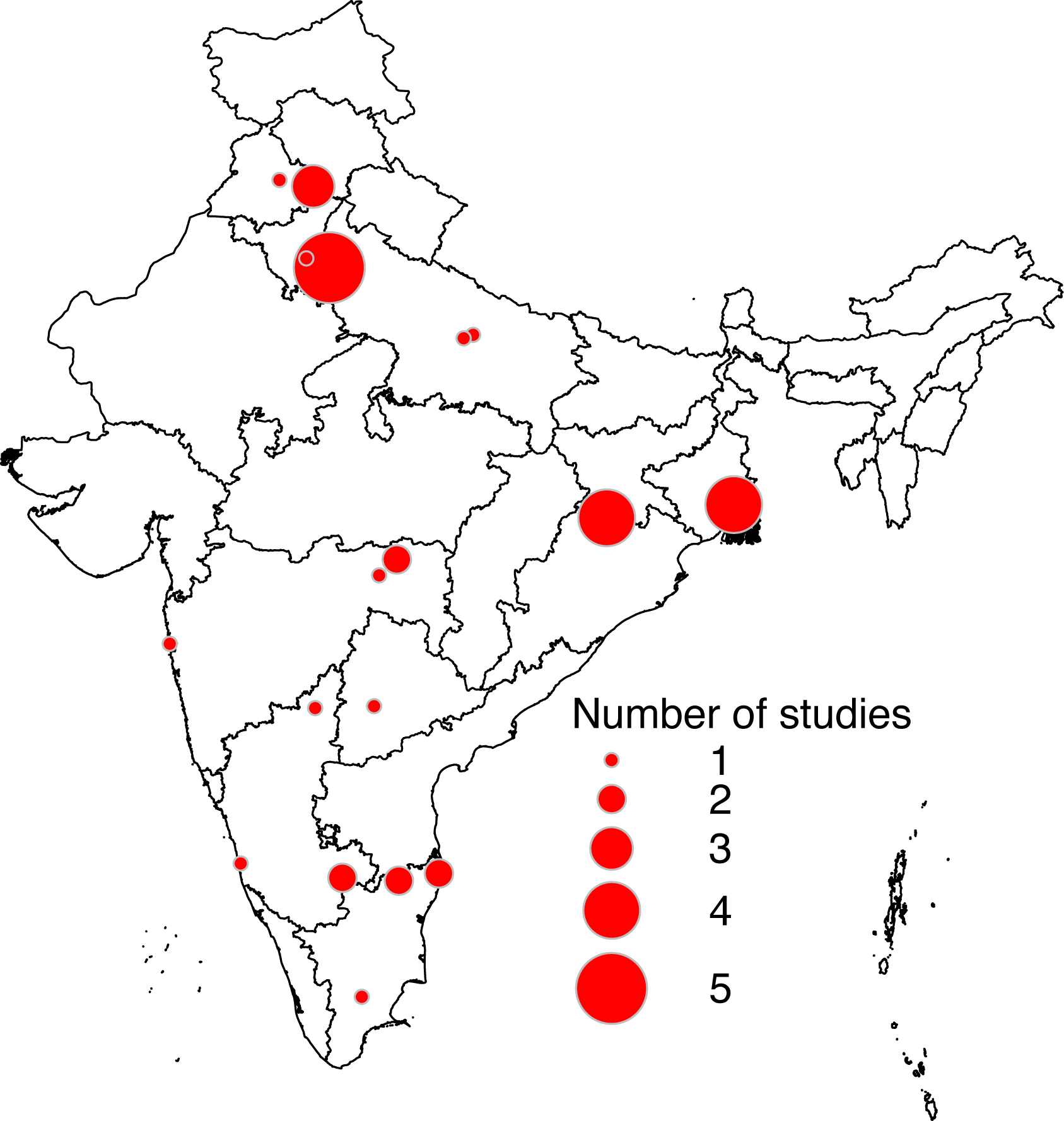

Supplement: S3 Fig — (TIF) [file pntd.0004616.s005.tif]
